# Effects of collimator angle, couch angle, and starting phase on motion‐tracking dynamic conformal arc therapy (4D DCAT)

**DOI:** 10.1002/acm2.12132

**Published:** 2017-07-21

**Authors:** Zhengzheng Xu, Rutao Yao, Matthew B. Podgorsak, Iris Z. Wang

**Affiliations:** ^1^ Department of Radiation Medicine Roswell Park Cancer Institute Buffalo NY USA; ^2^ Department of Nuclear Medicine State University of New York at Buffalo Buffalo NY USA; ^3^ Department of Physiology and Biophysics State University of New York at Buffalo Buffalo NY USA

**Keywords:** DCAT, MLC, motion tracking

## Abstract

**Purpose:**

The aim of this study was to find an optimized configuration of collimator angle, couch angle, and starting tracking phase to improve the delivery performance in terms of MLC position errors, maximal MLC leaf speed, and total beam‐on time of DCAT plans with motion tracking (4D DCAT).

**Method and materials:**

Nontracking conformal arc plans were first created based on a single phase (maximal exhalation phase) of a respiratory motion phantom with a spherical target. An ideal model was used to simulate the target motion in superior‐inferior (SI), anterior‐posterior (AP), and left‐right (LR) dimensions. The motion was decomposed to the MLC leaf position coordinates for motion compensation and generating 4D DCAT plans. The plans were studied with collimator angle ranged from 0° to 90°; couch angle ranged from 350°(−10°) to 10°; and starting tracking phases at maximal inhalation (θ=π/2) and exhalation (θ=0) phases. Plan performance score (PPS) evaluates the plan complexity including the variability in MLC leaf positions, degree of irregularity in field shape and area. PPS ranges from 0 to 1, where low PPS indicates a plan with high complexity. The 4D DCAT plans with the maximal and the minimal PPS were selected and delivered on a Varian TrueBeam linear accelerator. Gafchromic‐EBT3 dosimetry films were used to measure the dose delivered to the target in the phantom. Gamma analysis for film measurements with 90% passing rate threshold using 3%/3 mm criteria and trajectory log files were analyzed for plan delivery accuracy evaluation.

**Results:**

The maximal PPS of all the plans was 0.554, achieved with collimator angle at 87°, couch angle at 350°, and starting phase at maximal inhalation (θ=π/2). The maximal MLC leaf speed, MLC leaf errors, total leaf travel distance, and beam‐on time were 20 mm/s, 0.39 ± 0.16 mm, 1385 cm, and 157 s, respectively. The starting phase, whether at maximal inhalation or exhalation had a relatively small contribution to PPS (0.01 ± 0.05).

**Conclusions:**

By selecting collimator angle, couch angle, and starting tracking phase, 4D DCAT plans with the maximal PPS demonstrated less MLC leaf position errors, lower maximal MLC leaf speed, and shorter beam‐on time which improved the performance of 4D motion‐tracking DCAT delivery.

## 1 INTRODUCTION

Dynamic conformal arc therapy (DCAT) technique has been implemented in linear accelerator (linac) based stereotactic body radiotherapy (SBRT) for patients with Stage I/II non‐small‐cell lung cancer (NSCLC).^1^, ^2^, ^3^, ^4^, ^5^, ^6^, ^7^, ^8^, ^9^ One advantage of DCAT technique is the robust and transferable treatment methodology in planning, which is capable of reproducing the same or similar optimized planning results on different planning systems.^7^, ^8^, ^9^, ^10^ Compared to three‐dimensional (3D) conformal radiation therapy (3DCRT), DCAT has been proven to achieve higher target dose conformity and normal tissue dose sparing as well as shorter beam‐on time for dose delivery.^9^, ^10^ Rauschenbach et al.^10^ stated that DCAT should remain an alternative to 3DCRT in facilities that do not have VMAT or IMRT. Shi et al.^11^ have successfully implemented DCAT technique into clinical use for lung SBRT. They reported that the plan quality of DCAT met the RTOG protocols. Ouyang et al.^12^ reported that combining flattening filter free beams and DCAT provides promising improvements in NSCLC SBRT treatment in both plan quality and treatment planning efficiency. In addition, unlike VMAT, tumor coverage is not affected by MLC interplay effect.

Although hypo‐fractionated radiotherapy has demonstrated capability of providing high local control rates (85%–98%) in several phase I/II trials,^13^, ^14^, ^15^, ^16^, ^17^, ^18^, ^19^ blurred dose caused by tumor motion entails an increased risk of normal tissue toxicity.^20^, ^21^ Shimizu et al.^22^ reported lung tumor motion in SI direction was up to 24 mm. Several studies reported over 10 mm tumor motion in AP and LR directions.^23^, ^24^, ^25^ Zhao and colleagues^26^ reported dose deviation with motion is larger for smaller lung tumor (i.e., gross target volume is less than 10 cm^3^ in their study). Therefore, it is especially important to manage respiratory motion in hypo‐fractionated lung SBRT to ensure more accurate dose delivery. Tumor motion tracking is a recent development toward improving dose delivery quality. Compared with the common techniques of motion management such as respiratory gating and forced shallow breathing, motion‐tracking technique provides shorter treatment delivery time and requires less patient co‐operation and causes less patient discomfort.^27^, ^28^, ^29^

The effects of plan parameters in motion tracking have not been fully studied. Several studies implemented motion tracking with dynamic MLC treatment delivery for either Varian or Elekta linac and reported improved target dose coverage without significantly increasing the total treatment time.^30^, ^31^, ^32^, ^33^, ^34^, ^35^ Sawant et al. developed lung tumor motion compensation method where target motion that is decomposed to the beam's eye view (BEV) is dependent on collimator, gantry, and couch angles.^30^ Different combinations of collimator and couch angles will result in different tracking complexity which affects the delivery performance. Therefore, optimization of collimator and couch trajectories may reduce the possibility of having plans running at the mechanical limits of the linac, which can improve the treatment efficiency and robustness.^36^, ^37^ In most published studies on VMAT plans with motion tracking, collimator, and couch angles for plans were set at 90° and 0°, respectively, which has been shown to be favorable for MLC tracking because the MLC leaf motion direction is parallel to target motion in superior‐inferior (SI) direction.^30^, ^32^, ^34^, ^35^ However, for (3D) motion tracking, this collimator angle may not be the optimal solution for motion tracking.

In this study, we investigated the effects of collimator angle and couch angle on the performance, including MLC leaf position errors, MLC leaf speed, and total beam‐on time of DCAT plans with motion tracking (i.e., 4D DCAT). In addition, we also evaluated the effect of different starting tracking phases on 4D DCAT performance.

## 2 METHODS

### 2.A Respiratory three‐dimensional motion phantom and model

**Figure 1:**
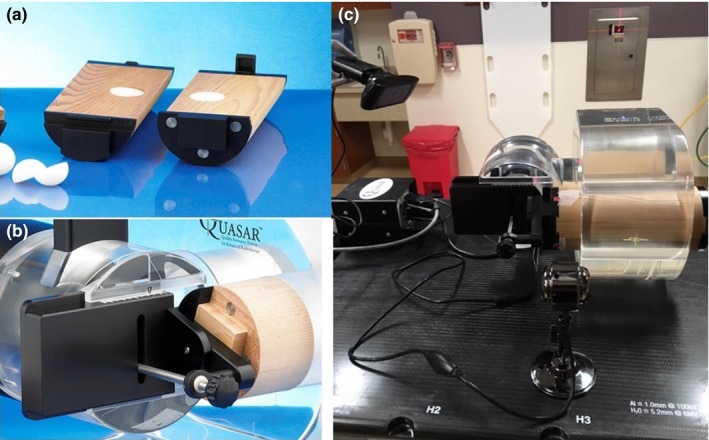
Respiratory motion phantom and setup. (a) Cedar insert with two off‐center hemispherical tumor phantoms. (b) The Cedar insert was hinged to the motor in the QUASAR phantom with a rotational stage to simulate 3D motion (photos were retrieved from http://modusqa.com/radiotherapy/phantoms/respiratory-motion, Modus Medical Device Inc., on October 22, 2016). (c) Two red markers were attached to the insert so that their positions could be tracked by two cameras.

**Figure 2:**
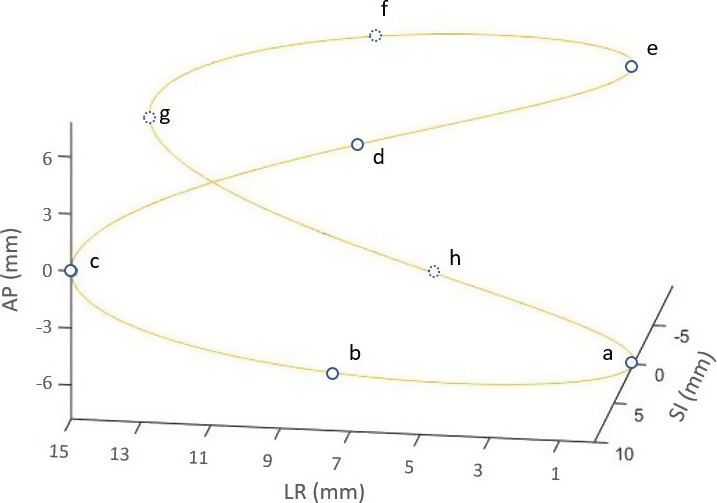
Illustration of spherical target motion according to the 3D rigid motion model. Blue circles represent the positions of target mass center at different time points. If the target starts to move from position “a,” then if follows the sequence of “a‐b‐c‐d‐e‐f‐g‐h‐a.”

The QUASAR^TM^ respiratory motion phantom (Modus Medical Device Inc., Canada) and a Cedar cylindrical insert with a 30 mm off‐centered spherical target (22 mm diameter) for simulating 3D respiratory motion was used in this study (Fig. 1). A rotational stage hinged the insert with the phantom motor which allows the target to rotate with 60^°^ of total motion range as it translates. As shown in Fig. 2, the target motion is the composite of reciprocating motion in the SI direction (z axis) and rotational motion in LR (left‐right, x axis) and AP (anterior‐posterior, y axis) plane. The target motion model is then given by

(1) $$z_{target}\left(t\right)=\frac{{A_{z}}^{\prime }}{2}\cos\left(\frac{2\pi t}{\tau }+\emptyset_{z}\right)$$

(2) $$y_{target}\left(t\right)=\rho \cdot \sin\left[\theta_{max}\cdot \cos\left(\frac{2\pi t}{\tau }+\emptyset_{y}\right)\right]$$

(3) $$x_{target}\left(t\right)=\rho \cdot \cos\left[\theta_{max}\cdot \cos\left(\frac{2\pi t}{\tau }+\emptyset_{x}\right)\right]$$

where ${A_{z}}^{\prime }$ **=** 20 mm was the peak‐to‐peak target motion amplitude, $\theta_{max}=\frac{\pi }{3}$ was the maximal rotational angle of the insert. The off‐center distance ρ was 30 mm. τ is the breathing cycle period and was set to 6 s. $\emptyset_{x}$, $\emptyset_{y}$, and $\emptyset_{z}$ are the starting phase for motion tracking, and ranged from maximal exhalation phases (∅=0) to maximal inhalation (∅=π/2).

### 2.B Generating 4D DCAT plans

#### 2.B.1 Nontracking DCAT Plans

All nontracking DCAT plans were generated based on CT images of a single respiratory phase (maximal exhalation phase), using Eclipse^TM^ treatment planning system (version 10.0, Varian Medical Systems, Inc., Palo Alto, USA), where collimator angle ranged from 0° to 90° with an increment of 1°, and couch angle ranged from 350° to 10° with an increment of 1°. Each plan was generated in the planning system with specific collimator and couch angle combination and exported as a DICOM plan file for motion compensation. Figure Figure A1A1 in Appendix A illustrates the collimator and couch angles in the treatment room setting. MLC leaves on both banks conformed to the contour of the stationary target during gantry rotation.

Each nontracking DCAT plan had 180 control points (CPs), with a full arc ranging from 181° to 179°. The collimator size (8 cm × 8 cm) was large enough to incorporate the target and its motion so that the collimator jaws would not block the beam during motion tracking. According to RTOG 0813, the prescription isodose surface for SBRT is normally chosen such that 95% of the PTV is conformably covered by the prescription isodose surface and 99% of the PTV receives a minimum of 90% of the prescription dose.^38^ For plans in this study, a simplified planning method was applied where 5 Gy (EBT3 film has high gradient response around 5 Gy) was prescribed to the isocenter set at the geometric center of the target with source to axis distance (SAD) of 100 cm.

#### 2.B.2 Motion‐tracking plans

The 4D DCAT plans were generated by applying the lung tumor motion‐tracking algorithm. The motion‐tracking method was based on *a priori* known rigid sinusoidal motion model that was projected to the BEV and compensated by MLC leaves.

For motion parallel to the direction of leaf travel, $MLC\left(m,n\right)$, the position of leaf “m” of CP “n” in the nontracking DCAT plan was transformed using:

(4) $$MLC{\left(m,n\right)}_{new}=MLC\left(m,n\right)+MLC_{\|}$$

where the motion compensation along MLC leaf travel direction, $MLC_{\|}$, is described in Appendix A. For motion perpendicular to the direction of leaf travel, all in‐field MLC leaves would be shifted laterally according to the motion direction by the following equation:

(5) $$\text{MLC}\;leaf\;pairs\;shift=\frac{{MLC}_{⊥}}{MLC\;leaf\;width}$$

where the motion compensation that is perpendicular to MLC leaf travel direction ($MLC_{⊥}$), is described in Appendix A. MLC leaves of both 2.5 mm and 5.0 mm width were involved in target motion compensation.

**Figure 3:**
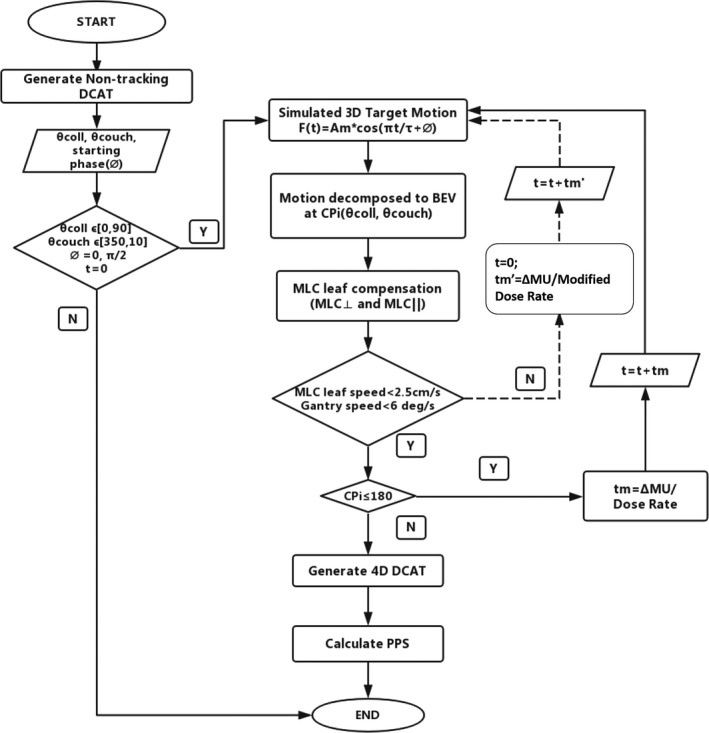
Flowchart of generating a deliverable 4D DCAT plan.

An in‐house developed MATLAB program was used for generating 4D DCAT plans with constant dose rate. The tracking algorithm was based on the algorithm developed by Sawant et al. for motion compensation^30^ and studies on modifications of beam parameters to generate a linac deliverable plan.^31^, ^33^, ^34^, ^35^ All machine parameters such as MLC leaf speed, gantry speed, and gantry acceleration were within limits (Table 1) after MLC leaf modification. As illustrated in Fig. 3, when leaf positions were modified for each CP, the speed of each MLC leaf and gantry speed were calculated using the maximal allowable dose rate. If there was any leaf speed exceeded the limit, the maximal dose rate would be reduced to the next available one and the leaf compensations for all CPs would be recalculated. Then, leaf speed would be calculated again based on new leaf positions and dose rate. The maximal allowable dose rate would be iteratively adjusted until all leaf speed at any specific CP was within the limit. As a result, all the MLC leaves involved in tracking could have enough time to reach to the planned position for motion compensation with constant dose rate.

**Table 1:** Machine constraints of the linear accelerator

Machine parameter	Maximal value^a^
Gantry speed (deg/s)	6.0
Gantry acceleration (deg/s^2^)	0.75
MLC leaf speed (mm/s)	25.0
Dose rate (MU/min)	600

a Reference: Varian Truebeam Datasheet.

### 2.C Plan Performance Score (PPS)

PPS was used to characterize the complexity degree of each 4D DCAT plan. PPS depends on the modulation complexity score (MCS),^39^ leaf travel index (LTI),^39^ and the maximal MLC leaf speed of a 4D DCAT plan with collimator angle at $\theta_{coll}$ and couch angle at $\theta_{ch}$ (eq. (6)). MCS focuses on the variability in MLC leaf positions, degree of irregularity in field shape, and area. These are related to the MLC aperture shape of each CP and leaf motion between adjacent CPs. As shown in Appendix A, motion projected to the BEV is dependent on collimator angle, couch angle, and starting phase. Therefore, PPS can be expressed as eq. (6).

(6) $$\begin{array}{cc} PPS\left(\theta_{coll},\theta_{ch},\emptyset \right) & =MCS\left(\theta_{coll},\theta_{ch},\emptyset \right)\cdot LTI\left(\theta_{coll},\theta_{ch},\emptyset \right)\cdot \\ & \left[1-\frac{\max\;\;MLC\;\;leaf\;\;speed\;\;of\;\;4D\;\;DCAT\;(\theta_{coll},\theta_{ch},\theta )}{Maximal\;\;MLC\;\;leaf\;\;speed\;\;limit}\right] \end{array}$$

MCS ranges from 0 to 1, and it approaches 0 for increasing degree of treatment plan complexity.^39^, ^40^, ^41^ LTI ranges from 0 to 1, and it approaches 0 for increasing total MLC leaf travel distance.^39^ PPS ranges from 0 to 1, a high PPS indicates that the plan of a specific configuration of collimator angle, couch angle, and starting phase has less degree of leaf position variability, aperture shape irregularity as well as low leaf speed.

A total of 3822 PPS values of 4D DCAT plans were calculated (91 collimator angles, 21 couch angles, and 2 starting tracking phases for 3D motion tracking). We selected the 4D DCAT plans with the minimal and the maximal PPS values, and corresponding nontracking DCAT plans for delivery experiments.

### 2.D Experimental delivery of 4D DCAT Plans

In the simulation algorithm, we assumed that dose rate and MLC leaves of each CP could instantaneously reach to planned values without any fluctuations. However, in actual delivery, there is a finite time before dose rate reaches the planned value.^31^, ^32^, ^35^ By acquiring delivery parameters (e.g., MLC leaf position errors) of 4D DCAT plans, we could evaluate the discrepancy between plans generated by simulation algorithm and the actual delivered ones. 4D DCAT plans were delivered using Varian TrueBeam^TM^ linac. TrueBeam trajectory log files were first analyzed for delivery parameter comparisons. The ArcCHECK^TM^ diode array (Sun Nuclear Corporation, Melbourne, USA) was also used to evaluate the accuracy of dose delivered by 4D DCAT plans. By comparing measurements to the calculated doses from treatment planning system (TPS) using the standard 3%/3 mm absolute dose gamma analysis, passing rates greater than 90% would imply that plan parameters have been properly transferred from control console computer to linac for delivery. All ArcCHECK^TM^ measurements were repeated on three consecutive days. The uncertainty was then obtained by evaluating the variation in repeated measurements.

All selected 4D DCAT plans were delivered to the QUASAR^TM^ respiratory motion phantom with the Cedar rotational insert. The rotational insert was able to provide rigid 3D target motion as demonstrated in eqs. (1), (2), (3). To ensure synchronization between target motion and 4D DCAT delivery, two high‐definition cameras were used to monitor the target position in real‐time. Two markers were attached to the insert for motion tracking [Fig. 1(c)]. An in‐house developed program was used to track the markers and display the positions of the markers in real‐time. Before each delivery, we calibrated the cameras and software to ensure the coordinates were consistent. When the tracking program was initiated, it focused on the markers and would change the tracking square color from yellow to red when the target reached to a specific breathing phase for beam initiation. In order to provide enough time for human response to manually turn on the beam, it would become yellow to green and remain for one‐second before it turned to red (i.e., the starting point for 4D DCAT delivery). During delivery, the verification system compared actual target position with the planned one. Once the discrepancy was higher than the tolerance (i.e., 1 mm), delivery would be paused in order to avoid significant de‐synchronization between MLC tracking and target motion.

Gafchromic EBT3 film was embedded in the target for dose measurement. All film pieces were cut in rectangular shapes congruent with the original sheet and scanned in landscape direction. In the scanned film image, the central axis on the film was first determined and a 3 cm by 3 cm square region of interest was selected (target diameter is 22 mm) to cover the whole measurement for gamma analysis. Planar dose of 4D DCAT plan delivered on the moving target phantom was compared to the nontracking DCAT plan delivered on the stationary target using gamma analysis using 3%/3 mm criteria. 4D DCAT plans were delivered three times for random error evaluation. By analyzing the exported data from trajectory log files, we compared MLC leaf speed, dose rate, root mean square error (RMSE) for MLC leaf deviations, and total beam‐on time between 4D DCAT plans.

$$\mathrm{RMSE}=\sqrt{\frac{\sum_{i=1}^{180}{\left(\bar{LP_{i}}-LP_{i}\right)}^{2}}{180}}$$

where $\bar{LP_{i}}$ and $LP_{i}$ were the actual and planned MLC leaf positions at CPi, respectively.

In an ideal motion‐tracking situation, dose delivered by 4D DCAT on the moving target should be the same as the dose delivered by the nontracking DCAT on the static target. MLC leaf position errors, which were recorded during 4D DCAT plan delivery, were applied to nontracking DCAT plan for dose calculation in order to evaluate its dosimetric effect on 4D DCAT delivery. Difference in minimal target dose ($\Delta D_{min}$) and maximal target dose ($\Delta D_{max}$) were calculated. Dose differences to normal organs such as spinal cord and lung were also evaluated.

## 3 RESULTS

### 3.A Effects of collimator angle, couch angle, and starting phase on PPS

**Figure 4:**
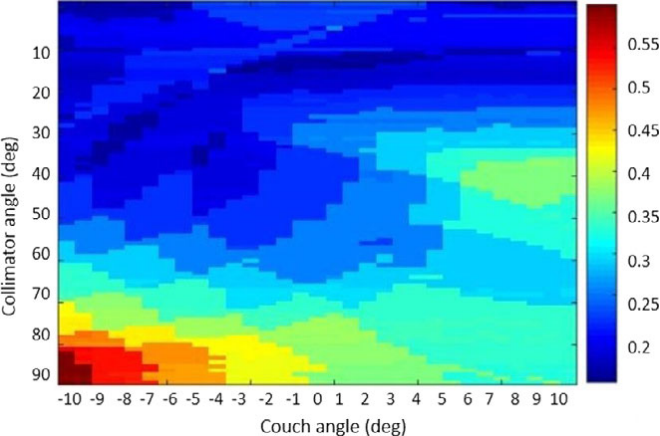
The effect of collimator and couch angle configuration on PPS of 4D DCAT plans. Each pixel value represents the PPS of a 4D DCAT plan with specific collimator and couch angles combination.

With the combinations of 91 collimator and 21 couch angles, the mean and standard deviation of PPS values of nontracking DCAT plans were 0.996 and 0.002, respectively. The small variation in PPS values demonstrated similar degrees of plan complexity for nontracking DCAT plans with different collimator and couch angles. Figure 4 demonstrates the effects of collimator and couch angle combination on the plan complexity for 4D DCAT with 3D motion tracking. The 4D DCAT plan with the maximal PPS (0.554) was acquired when collimator and couch were at 87° and 350° (−10°), respectively. The plan with the minimal PPS (0.197) was acquired when collimator and couch were at 15° and 0°, respectively. The difference in PPS between 4D DCAT with starting phase at maximal inhalation and those with starting phase at maximal exhalation was 0.01 ± 0.05.

### 3.B Quality of experimentally delivered 4D DCAT Plans

**Figure 5:**
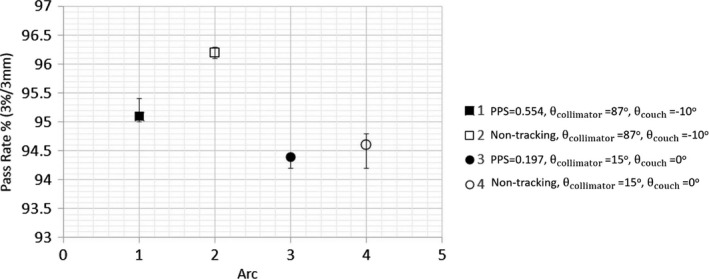
Passing rates of ArcCHECK measurements of each arc in 4D DCAT plans and nontracking DCAT plans.

Figure 5 demonstrates the ArcCHECK^TM^ measured passing rate (3%/3 mm criteria and global maximum dose normalization) of each arc in nontracking DCAT and 4D DCAT plans with the maximal and minimal PPS values, and the variation was evaluated based on repeated measurements on three consecutive days. The maximal variation found was 0.5% with respect to 94.6% average passing rate.

**Figure 6:**
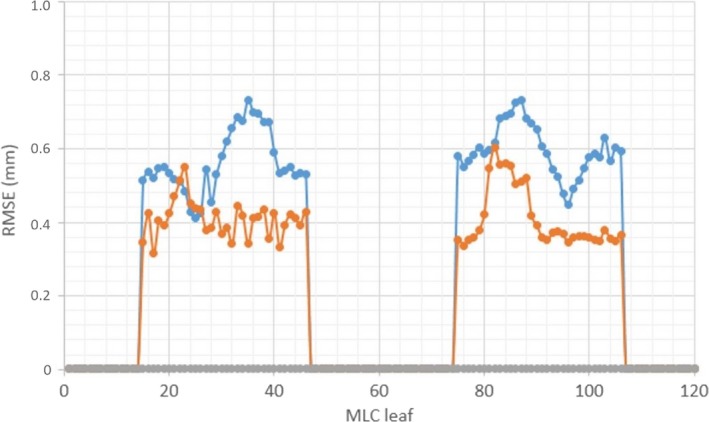
RMSE of MLC leaves (1–120) for 4D DCAT plans. Blue: 4D DCAT with the minimal PPS (0.197). Orange: 4D DCAT with the maximal PPS (0.554). Gray: nontracking DCAT plans (PPS≈1).

**Figure 7:**
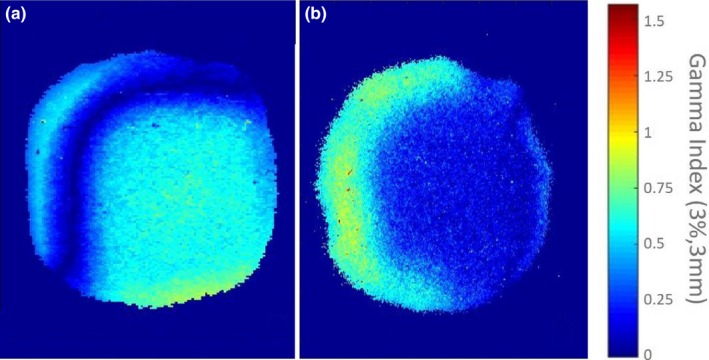
An example of gamma analyses for film measurements of 4D DCAT plans. (a) Passing rates of 4D DCAT plan with the maximal PPS: 99.1%. (b) Passing rates of 4D DCAT plan with the minimal PPS: 97.1%. Failed points are those pixels in the figure with gamma index >1.

**Figure 8:**
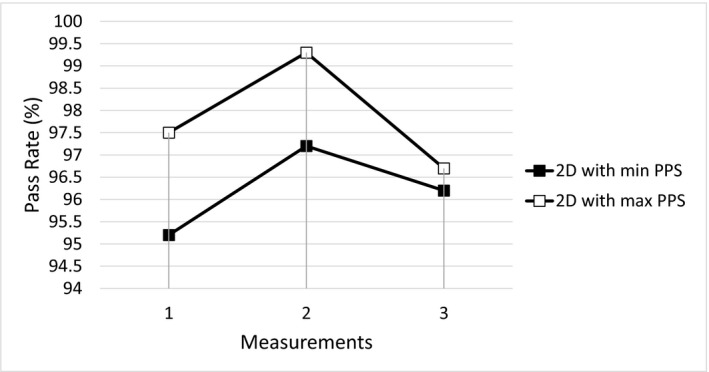
Gamma Passing rates of three repeated film measurements of 4D DCAT plans. Soft square: 4D with the maximal PPS; Solid square: 4D DCAT with the minimal PPS.

For 4D DCAT with the maximal PPS, the maximal MLC leaf speed, total leaf travel distance, MLC leaf RMSE, and total beam‐on time were 20 mm/s, 1385 cm, and 157 s, respectively. Compared to 4D DCAT with the minimal PPS, plan with the maximal PPS demonstrated improved motion‐tracking performance including smaller MLC leaf errors (Fig. 6), lower maximal MLC leaf speed, shorter total leaf travel distance, and shorter total beam‐on time (Table 2). The 4D DCAT with the maximal PPS also allowed higher dose rate (298.1 MU/min) for motion tracking. In addition, passing rates of film measurements for 4D DCAT plans with the maximal PPS demonstrated higher passing rates (97.7% ± 1.0%) as shown in Figs. 7 and 8.

**Table 2:** Parameters of 4D DCAT plans with the maximal and minimal PPS values

Parameters	4D DCAT Plans	
PPS value	**0.554**	0.197
Collimator (deg)	**87°**	15°
Couch (deg)	**350°(−10°)**	0°
Maximum leaf speed (mm/s)	**20**	25
MLC leaf errors (mm)	**0.39 ± 0.16**	0.57 ± 0.11
Total beam‐on time (s)	**157**	239
Total leaf travel distance (cm)	**1385**	1713
Average dose rate (MU/min)	**298.1 ± 0.4**	196.1 ± 2.0

**Figure 9:**
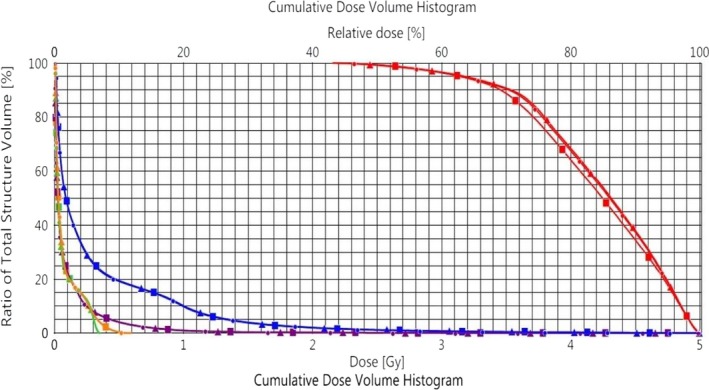
Evaluation of dosimetric effect caused by MLC errors during tracking. Circular: nontracking DCAT plan. Triangular: nontracking DCAT plan with MLC errors from 4D DCAT (PPS = 0.554) delivery; Square: nontracking DCAT plan with MLC errors from 4D DCAT (PPS = 0.197) delivery. Red lines: target. Blue lines: left lung. Green lines: spine. Purple lines: body. Orange lines: heart.

Dosimetric differences in Dmin and Dmax to the target between 4D DCAT and nontracking DCAT plans were 0.02 Gy and 0.04 Gy for 4D DCAT with the maximal PPS, and 0.11 Gy and 0.06 Gy for 4D DCAT with the minimal PPS, respectively (Fig. 9 and Table 3). Differences in dosimetric indices to the organs such as spinal cord and lung are also listed in Table 3. There was a minimal difference in heart doses between the two 4D DCAT plans.

**Table 3:** Dosimetric effect of MLC leaf position errors on 4D DCAT plans

PPS	^+^∆Dmin (Gy)	^+^∆Dmax (Gy)			
	Target	Target	Spinal cord	Body	Lt. lung
0.554	−0.02	−0.04	0.00	0.00	0.00
0.197	−0.11	−0.06	0.03	0.01	0.01

^+^Positive value means the structure dose of a 4D DCAT plan with MLC errors is higher than that of the nontracking DCAT plan.

## 4 DISCUSSION

Ideally, for a nontracking DCAT plan, when the isocenter is at the target geometric center and the target shape is symmetrical, the target projection to the MLC coordinates at each CP should be identical. In this case, the ideal PPS for nontracking DCAT plans with different collimator and couch angles should be equal to one. In reality, since the target contour generated in the TPS was not a perfect sphere, it resulted in the variation in target projection at each CP (i.e., slight MLC shape variation at each CP). Therefore, PPS results were slightly less than one but also very close to one among different nontracking plans in the study. For the 4D DCAT plans, on the other hand, the PPS values were significantly smaller than one, with a best score of 0.554 in the study. It indicated an increased complexity when carrying out motion tracking using dynamic MLC technique. Compared to the optimization results using 1° gantry angle increment (maximal PPS: 0.554), when using 2° and 5° increments for optimizations, the maximal PPS of 4D DCAT plans were 0.554 and 0.543, respectively. Therefore, similar optimization results can be achieved with higher gantry angle increment (e.g., <5° in this study) which reduces the total optimization time.

The error bars in Fig. 5 show relatively small variation in measurement of delivered plans, which indicates that all plan parameters had been correctly transferred from control console computer to linac for delivery. High passing rates of film measurements and small RMS of MLC leaf position errors verified the accuracy of motion‐tracking simulation algorithm. Compared to 4D DCAT with the minimal PPS, plan with the maximal PPS showed higher average dose rate and less MLC leaf position errors which resulted in faster and more accurate motion tracking. Because of the ideal target shape and motion model, the leaf errors did not significantly affect the passing rates and target dose coverages of 4D DCAT plans with the maximal and minimal PPS. For an irregular shaped tumor with more complicated motion pattern, one would expect the beam aperture difference and target motion between adjacent CPs be larger. Therefore, plan with the minimal PPS will be expected to have more leaf errors during tracking since more leaves will be moving at the maximal speed.

Passing rates of all the film measurements are higher than 90% threshold when using 3%/3 mm criteria, which demonstrates the reliability of the synchronization method using manual beam initiation and cameras for target motion monitoring. Failed points (i.e., yellow and red points) in Fig. 7 indicate motion blurring caused by de‐synchronization during dose delivery.

Using lasers, phantom markers, and digital level for phantom alignment, the measurement setup was of high consistency. Since there is no external control for MLC leaves, motion tracking cannot be interrupted to correct for any discrepancy during delivery. The motion‐tracking accuracy is sensitive to the synchronization between beam initiation and target motion. There are several factors that affect synchronization during 4D DCAT delivery. First, for each 4D DCAT plan delivery, there was a difference in human reaction delay in beam initiation when the target reached to its planned position. Then, linac requires time to reach to the planned dose rate when beam is initiated. Thirdly, with the existence of MLC motor problems and friction between adjacent leaves, some leaves at specific CPs were unable to move at the maximal speed (25 mm/s) to catch up with the target motion. Therefore, linac had to reduce the dose rate in order to move those leaves to the planned position in time. In addition, during tracking, the linac demonstrated higher dose rate instability at lower dose rate level. Because of these effects, MLC leaves were unable to accurately catch the motion as predicted in the tracking simulation. One solution is to reduce the maximal leaf speed limit to a lower level in motion‐tracking algorithm so that MLC leaves can move more smoothly without affecting dose rate. One disadvantage of slowing down leaf speed is that the total delivery time will be longer.

In addition to de‐synchronization, motion‐tracking accuracy is affected by the tracking method for motion perpendicular to the leaf travel direction. Since the MLC leaf width in the central 8 cm field is 2.5 mm, the motion will not be compensated if the amplitude is less than 2.5 mm.

Falk and colleagues^28^ recently reported on the delivery efficiency of motion‐tracking plan. They pointed out an increased treatment time could potentially increase the risk of systematic drifts of the tumor position. In this study, total beam‐on time of 4D DCAT with the maximal PPS (157 s) was less than the plan with the minimal PPS (239 s), which potentially reduces the possibility of introducing tumor motion variability during delivery.

The main purpose of this study was, by introducing a PPS‐based motion‐tracking algorithm, one can improve the tracking performance of a 4D DCAT plan by optimizing the configuration of collimator and couch angles. All the results demonstrated are based on a phantom with an ideal rigid motion pattern. It is not intended as a report on the improvement of motion management ready for clinical treatment. Based on current work, future studies will involve using optimization algorithm to select the combination of collimator and couch angles for 4D DCAT plans with irregular shaped target and/or actual patient breathing motion.

## 5 CONCLUSION

In this study, by active selection of collimator angle and couch angle, one can achieve a 4D DCAT plan with the maximal PPS. The 4D DCAT with the maximal PPS demonstrated improved motion‐tracking performance including less MLC leaf position errors, lower maximal MLC leaf speed and shorter beam‐on time. Starting tracking phase has a small impact on the 4D DCAT plan performance.

## Figures and Tables

**Figure Figure A1:**
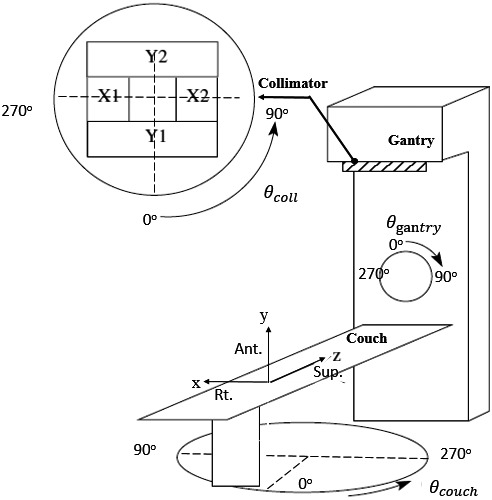
Illustration of room coordinates in motion tracking.

## Appendix A

### Implementation of MLC Tracking

$MLC_{\|}$was the projected motion that is parallel to the MLC leaf travel direction.

(A1) $$\begin{array}{cc} MLC_{\|} & ={\left[\begin{array}{c} cos\theta_{ch}\cdot sin\theta_{coll}-sin\theta_{ch}\cdot cos\theta_{coll}\cdot cos\theta_{gan}\left(n\right)\\ cos\theta_{coll}\cdot sin\theta_{gan}\left(n\right)\\ -cos\theta_{ch}\cdot sin\theta_{coll}-cos\theta_{ch}\cdot cos\theta_{coll}\cdot cos\theta_{gan}\left(n\right) \end{array}\right]}^{T}\\ & \;\cdot \left[\begin{array}{c} z_{target}\left(t=\sum_{m=1}^{CP}t_{m}\right)\\ y_{target}\left(t=\sum_{m=1}^{CP}t_{m}\right)\\ x_{target}\left(t=\sum_{m=1}^{CP}t_{m}\right) \end{array}\right]\cdot M\_factor \end{array}$$

where motion elements in superior, anterior, and right directions are positive; motion elements in inferior, posterior, and left are negative. $\theta_{coll}$ is the collimator angle, $\theta_{ch}$ is the couch angle, and $\theta_{gan}$ is the gantry angle at CP “n” (Fig. Figure A1A1), where n ranged from 0 to 180. $t_{m}$ is the time segment between adjacent CPs, given by

(A2) $$t_{m}=\frac{\Delta \text{MU}}{Dose\;\;Rate}$$

ΔMU=Total MU×MU weight factor

where ΔMU is the MU between two adjacent CPs.

Magnification caused by motion parallel to the beam axis is corrected by the *M_factor*.

(A3) $$MLC_{CAX}={\left[\begin{array}{c} sin\theta_{ch}\cdot sin\theta_{gan}\left(n\right)\\ cos\theta_{gan}\left(n\right)\\ cos\theta_{ch}\cdot sin\theta_{gan}\left(n\right) \end{array}\right]}^{T}\cdot \left[\begin{array}{c} z_{target}\left(t=\sum_{m=1}^{CP}t_{m}\right)\\ y_{target}\left(t=\sum_{m=1}^{CP}t_{m}\right)\\ x_{target}\left(t=\sum_{m=1}^{CP}t_{m}\right) \end{array}\right]$$

(A4) $$M_{\mathrm{factor}}=\left[\frac{100\;-\;{\mathrm{MLC}}_{CAX}}{100}\right]$$

where 100 cm is the SAD.

For motion that is perpendicular to the direction of leaf travel, all in‐field MLC leaves will be shifted laterally according to the motion direction by the following equation:

(A5) $$\text{MLC leaf pairs shift}=\frac{{\text{MLC}}_{⊥}}{\text{MLC leaf width}}$$

${\text{MLC}}_{⊥}$ is the projected motion perpendicular to the MLC leaf travel direction.

(A6) $$\begin{array}{cc} MLC_{⊥} & ={\left[\begin{array}{c} cos\theta_{ch}\cdot cos\theta_{coll}+sin\theta_{ch}\cdot sin\theta_{coll}\cdot cos\theta_{gan}\left(n\right)\\ -sin\theta_{coll}\cdot sin\theta_{gan}\left(n\right)\\ -sin\theta_{ch}\cdot cos\theta_{coll}+cos\theta_{ch}\cdot sin\theta_{coll}\cdot cos\theta_{gan}\left(n\right) \end{array}\right]}^{T}\\ & \;\cdot \left[\begin{array}{c} z_{target}\left(t=\sum_{m=1}^{CP}t_{m}\right)\\ y_{target}\left(t=\sum_{m=1}^{CP}t_{m}\right)\\ x_{target}\left(t=\sum_{m=1}^{CP}t_{m}\right) \end{array}\right]\cdot M\_factor \end{array}$$
